# Chronic Kidney Disease in Metabolic Disease: Regulation of SGLT2 and Transcriptomic–Epigenetic Effects of Its Pharmacological Inhibition

**DOI:** 10.3390/ijms27020589

**Published:** 2026-01-06

**Authors:** Chiara Salvà, Susanne Kaser, Matteo Landolfo

**Affiliations:** 1 Department of Internal Medicine I, Medical University of Innsbruck, 6020 Innsbruck, Austria; chiara.salva@student.i-med.ac.at (C.S.); m.landolfo@staff.univpm.it (M.L.); 2 Clinical and Molecular Sciences Department, University Politecnica delle Marche, 60126 Ancona, Italy

**Keywords:** sodium–glucose cotransporter 2, chronic kidney disease, cardio-nephro-metabolic syndrome, metabolic disease, transcriptomics, epigenetics, SGLT2 inhibitors

## Abstract

Sodium–glucose cotransporter 2 inhibitors (SGLT2is) have revolutionized the management of type 2 diabetes mellitus, heart failure, and chronic kidney disease (CKD), providing cardiorenal and metabolic benefits that extend beyond glycemic control. While their clinical efficacy is well established, the underlying molecular mechanisms remain only partially understood. This review focuses on current knowledge of SGLT2 expression and regulation in health and metabolic diseases, as well as transcriptional and epigenetic consequences of pharmacological SGLT2 inhibition. Human and experimental studies demonstrate that SGLT2 expression is confined to proximal tubular cells and regulated by insulin, the renin–angiotensin–aldosterone system, the sympathetic nervous system, oxidative stress, and transcriptional and epigenetic pathways. SGLT2 expression follows a biphasic pattern in metabolic disorder-associated CKD: upregulation in early phases and reduction in advanced stages. Evidence from animal models and single-cell transcriptomic studies indicates that SGLT2is normalize metabolic and inflammatory gene networks. To our knowledge, a recent single-cell RNA sequencing study provides the only currently available human dataset linking SGLT2i therapy with tubular metabolic rewiring and suppression of the energy-sensitive mechanistic target of rapamycin complex 1. Collectively, these findings support a model in which SGLT2 inhibition mitigates metabolic stress by restoring energy homeostasis across multiple nephron segments.

## 1. Introduction

Sodium–glucose cotransporter 2 inhibitors (SGLT2is) have transformed the therapeutic landscape of type 2 diabetes mellitus (T2DM), chronic kidney disease (CKD), and heart failure. Large cardiovascular and renal outcome trials in patients with and without T2DM have consistently demonstrated reductions in cardiovascular morbidity and mortality, hospitalization for heart failure, and progression of CKD [1,2]. Regarding primary renal outcomes, major clinical trials have shown beneficial effects in patients with T2DM and CKD (e.g., the CREDENCE trial), including a lower risk of end-stage kidney disease and of kidney function decline [3].

Further studies have shown that SGLT2i therapy decreases the risk of kidney disease progression in patients with and without diabetes, as demonstrated in the EMPA-KIDNEY and DAPA-CKD trials [4,5]. These results were confirmed by subsequent meta-analyses, emphasizing the use of SGLT2is in patients with CKD, irrespective of diabetic status, kidney function, or primary kidney disease, and demonstrating reduced renal outcomes, independent of the albuminuria level [6,7,8,9]. SGLT2is have also shown benefits beyond glycemic control in children and adolescents, with data indicating potential renoprotective effects, as explained in detail in a recent review [10]. However, evidence in these populations remains limited, and potential long-term effects on growth and development need to be addressed in future studies. Caution is warranted when extrapolating and adapting findings from adult populations to children.

Within the kidneys, the benefits of SGLT2is extend far beyond glucose lowering and moderate weight reduction, likely arising from a constellation of metabolic and hemodynamic mechanisms. Established renoprotective pathways include natriuresis, intraglomerular pressure reduction through restoration of tubuloglomerular feedback, enhanced cellular energetics, and modulation of inflammation and oxidative stress pathways [11,12]. Additionally, as extensively described in recent reviews, by initiating systemic cascades, SGLT inhibition acts way beyond its primary renal effects, involving systemic hemodynamic, structural, and metabolic changes that eventually lead to the reduction in interstitial volume, which improves cardiac function, to beneficial shifts in energy metabolism, and to the amelioration of inflammation and fibrosis. These therapeutic agents also reduce afferent renal nerve signaling, consequently decreasing sympathetic nervous system (SNS) activity, a mechanism that provides a fundamental contribution to overall cardiometabolic and renal protection [13].

Despite the wide use of SGLT2is in clinical practice, the regulation of SGLT2 expression is not completely understood. Growing evidence indicates that renal SGLT2 expression and activity do not follow a linear trajectory but instead display a biphasic pattern shaped by the hormonal, metabolic, and epigenetic perturbations associated with cardiovascular and metabolic diseases. Consequently, although SGLT2is provide substantial clinical benefits, and several mechanisms of action have been proposed and validated, the precise molecular and cellular pathways that confer kidney protection remain incompletely defined, particularly in advanced CKD, where SGLT2 expression is markedly reduced [14,15].

Recent transcriptomic analyses further suggest that pharmacologic SGLT2 blockade induces widespread transcriptional remodeling within the kidneys, underscoring the complexity of its renoprotective effects and highlighting the need for continued mechanistic investigation [16]. This review provides an overview of the current knowledge on renal SGLT2 expression and regulation in health and metabolic diseases, integrating data from human studies and animal models. It also highlights emerging transcriptomic evidence on SGLT2i-mediated gene expression changes and incorporates insights from genomic and epigenomic analyses using methodologies beyond quantitative polymerase chain reaction (PCR) and single-gene approaches—such as RNA sequencing and DNA methylation profiling—which provide an unbiased, systems-level perspective on how SGLT2 inhibition reshapes the molecular landscape of the diseased kidney.

## 2. SGLT2 Expression and Regulation in the Proximal Tubule

SGLT2, encoded by SLC5A2 on chromosome 16p12–p11, is the principal mediator of glucose reabsorption in the kidneys. Foundational biochemical and molecular studies have established that SGLT2 expression is restricted to the S1 and S2 segments of the proximal convoluted tubule (PT), where it reclaims approximately 90% of filtered glucose [17,18]. Loss-of-function mutations in SLC5A2 result in familial renal glycosuria, underscoring the essential role of the protein in renal glucose handling and systemic glucose homeostasis [19].

Recent advances in omics and spatial profiling technologies have refined this understanding. Transcriptomic and proteomic atlases consistently identify the kidneys as the organ with the highest SLC5A2 expression, while single-cell transcriptomic and chromatin accessibility studies further delineate promoter-level regulatory features that distinguish SGLT2-expressing from SGLT1-expressing nephron segments [20].

These insights deepen the molecular framework of renal glucose transport. Importantly, conventional immunocytochemical and molecular assays provide information about expression and localization but may not accurately reflect protein abundance or transporter activity. SGLT2 function is determined not only by protein quantity but also by turnover dynamics and post-transcriptional regulation, underscoring the need for integrated functional assessments [21].

In both physiological and pathological states, SGLT2 expression and activity are dynamically modulated by hormonal and metabolic cues [22]. Bioinformatic analyses have identified the expression of SGLT2 network components in several tissues, including the kidneys, liver, adipose tissue, blood, and heart, and have revealed interactions with key metabolic and signaling pathways, involving sirtuin-1, adiponectin, insulin, glucose transporter 4, glucose transporter 1, and angiotensin-converting enzyme [23]. Recent reviews have also highlighted the contributions of non-coding RNAs in shaping this regulatory landscape, with potential implications for disease mechanisms and therapeutic targeting [24]. Epigenetic processes—including chromatin remodeling and nucleosome positioning—further modulate promoter accessibility, providing a mechanistic link through which metabolic and inflammatory inputs can fine-tune SGLT2 expression [25].

Collectively, these multilayered regulatory mechanisms integrate local PT function with systemic metabolic status, supporting the concept that SGLT2 is not a static transporter but a component of a dynamic metabolic-sensing network. This complexity may help explain why SGLT2is exert pleiotropic effects that extend well beyond the inhibition of glucose reabsorption.

### 2.1. Biphasic Trajectory of SGLT2 Expression in Metabolic Disorder-Associated CKD

**Figure 1:**
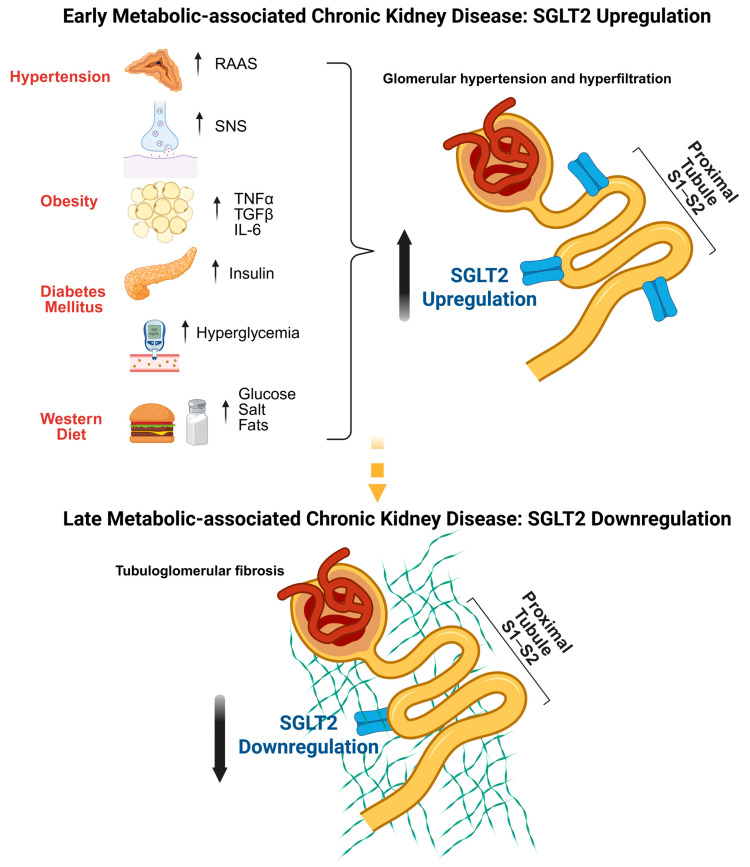
**Sodium–glucose cotransporter 2 (SGLT2) trajectory in metabolic disorder-associated CKD.** Progression from early metabolic disorder-associated CKD, characterized by upregulation of SGLT2 driven by dysmetabolic and neurohormonal stimuli, to late stages of metabolic disorder-associated CKD, characterized by tubuloglomerular fibrosis, tubular dedifferentiation, and reduced SGLT2 expression. IL-6: interleukin-6; RAAS: renin–angiotensin–aldosterone system; SNS: sympathetic nervous system; TGF-β: transforming growth factor-β; TNF-α: tumor necrosis factor-α.

Metabolic syndrome, obesity, T2DM, and hypertension are well-established risk factors for the development of CKD, particularly in the context of metabolic disorder-associated CKD. Histopathological studies have identified characteristic—and, in some cases, distinct—patterns of kidney injury associated with these conditions, particularly involving the glomeruli [26]. Despite their differences, a unifying feature is the progression from an early adaptive–maladaptive phase characterized by PT hypertrophy and glomerular hyperfiltration to a state of accumulating interstitial fibrosis, oxidative stress, and low-grade inflammation, ultimately culminating in glomerulosclerosis and kidney failure [27], as presented in Figure 1.

Excess caloric and sodium intake trigger the development of metabolic syndrome, obesity, T2DM, and hypertension by inducing or aggravating insulin resistance, leading to hyperinsulinemia, chronic exposure to elevated glucose and lipid levels, and overactivation of the SNS and the renin–angiotensin–aldosterone system [28]. These shared dysmetabolic stressors directly and indirectly promote upregulation of renal SGLT2 expression and activity, a hallmark of the early stages of CKD in metabolic diseases.

#### 2.1.1. Hyperinsulinemia and Hyperglycemia

Elevated insulin levels stimulate SGLT2 expression through intracellular signaling pathways, including protein kinase A- and C-dependent phosphorylation [29], as well as through the direct activation of exchange proteins (Epac) by the 3′,5′-cyclic adenosine monophosphate (cAMP) signaling pathway, which is also implicated in the regulation of renal water handling, sodium and potassium transport, and PT metabolic responses [30]. Chronic hyperglycemia further enhances SGLT2 expression and promotes its translocation to the apical membrane, again via protein kinase A-dependent pathways [31]. This upregulation following high-glucose exposure can be attenuated by the hormone gastrin through the renal cholecystokinin B receptor (CCKBR) via the Erk/nuclear factor-kappa B (NF-κB) pathway. In a previous study, under diabetic conditions, CCKBR was downregulated, and knockout mice showed increased susceptibility to obesity and diabetes under a high-fat diet [32].

#### 2.1.2. Pro-Fibrotic and Inflammatory Signaling

Hypercaloric diets and high-glucose environments at the PT level promote the local release of pro-fibrotic and inflammatory cytokines, such as transforming growth factor-β, interleukin-6, and tumor necrosis factor-α, which upregulate SGLT2 mRNA and protein expression, creating a self-perpetuating vicious cycle in which increased glucose influx enhances the production of transforming growth factor-β and promotes fibrosis [33]. Chronic high basolateral glucose exposure in the PT also induces nuclear translocation of hepatocyte nuclear factor-1α, a key transcriptional activator of SLC5A2, and suppresses SIRT1, a deacetylase with well-described antioxidative and anti-inflammatory properties [34].

#### 2.1.3. Neurohumoral Activity

Overactivity of the renin–angiotensin–aldosterone system and the SNS has been associated with increased SGLT2 expression [35,36]. Kidney biopsy studies report positive correlations between SGLT2 expression and intrarenal renin–angiotensin–aldosterone system components, while experimental data demonstrate that angiotensin II (Ang II) upregulates SGLT2 via Ang II receptor type 1 receptor activation and oxidative stress. In Ang II-induced hypertensive animal models, SGLT2 levels are elevated and accompanied by reduced kidney function and proteinuria [35]. Noradrenaline similarly increases SGLT2 expression in cultured PT cells, and chemical sympathectomy in hypertensive mice reduces both blood pressure (BP) and SGLT2 expression, linking transporter regulation to SNS tone [36]. In hypertensive mice, chemical denervation of the SNS lowers BP and significantly reduces fasting glucose levels and SGLT2 expression [37].

#### 2.1.4. Excessive Dietary Lipids and Salt

In diet-induced obesity, renal lipid accumulation is associated with higher SGLT2 expression and augmented glucose reabsorption, linking altered lipid handling to transporter regulation [38]. Sodium intake also modulates SGLT2 via adipokine-mediated and Y-box binding protein-1 (YB-1)-associated pathways [39,40]. High salt intake upregulates peroxisome proliferator-activated receptor-δ (PPARδ) expression, promoting adiponectin release, which, in turn, suppresses SGLT2 transcription. In dysmetabolic states, adiponectin deficiency weakens this inhibitory loop, allowing sodium-driven neurohormonal activation to promote SGLT2 upregulation in the PT [39]. Y-box binding protein-1, whose levels increase under high-sodium conditions, reduces translation of SGLT2 mRNA, adding one more layer of post-transcriptional regulation [40].

Human genetic data further support the role of SGLT2 in BP regulation, with SLC5A2 polymorphisms associated with salt sensitivity, BP variability, and incident hypertension [41]. Recent single-cell RNA sequencing (scRNA-seq) studies in youth-onset T2DM corroborate these findings, showing early increases in SLC5A2 expression [42]. As the disease progresses, however, SGLT2 expression typically declines. This downregulation parallels worsening fibrosis, loss of tubular structural integrity, and the transition from a hyperfunctional to a dedifferentiated and ultimately atrophic state in advanced CKD [43].

Several additional pathways modulate SGLT2 in this context. The multiligand endocytic receptor megalin influences SGLT2 abundance, as megalin-knockout mice exhibit downregulated SLC5A2 transcripts and reduced SGLT2 protein expression. These animals display improved glucose tolerance when fed a Western diet but develop more severe interstitial fibrosis, suggesting that reduced SGLT2 expression can coexist with progressive structural kidney injury [44]. Transcriptome-wide analyses further support this concept, showing decreased SLC5A2 expression in advanced metabolic disorder-associated CKD and strong associations with fibrosis-related gene programs [45].

These observations support the concept that SGLT2 expression reflects both metabolic burden and tubular structural health. Increased SGLT2 levels in early disease may represent a maladaptive response to glycosuria and sodium stress, whereas progressive fibrosis, inflammatory injury, and cellular stress ultimately suppress transporter abundance. The variability in findings underscores the need for stage-specific, cell-resolved, and longitudinal analyses to fully delineate SGLT2 dynamics across the spectrum of CKD in metabolic disease.

## 3. Transcriptional and Epigenetic Effects of SGLT2 Inhibition in the Kidneys

SGLT2is have demonstrated broad beneficial effects at the renal level—via both glucose control and hemodynamic modulation as well as by consistently inducing metabolic shifts that exert anti-inflammatory and anti-fibrotic actions—highlighting their growing clinical relevance and therapeutic potential [13,46]. In this context, accumulating evidence demonstrates that SGLT2is exert substantial gene-regulatory effects in the kidney, influencing pathways involved in metabolism, inflammation, and fibrosis [47,48].

Until recently, most insights into SGLT2-related gene regulation in the kidneys were derived from candidate-gene approaches using quantitative PCR, which are inherently limited in scope and unable to capture cell-type-specific responses. Earlier experimental work in human PT cells using targeted real-time quantitative polymerase chain reaction (RT-qPCR) approaches had already suggested that SGLT2 inhibition downregulates pro-inflammatory and pro-fibrotic gene expression while preserving epithelial phenotype and supporting an anti-inflammatory action at the tubular level [49]. These candidate-gene data are now complemented and extended by unbiased single-cell transcriptomic profiling in human tissue, which delineates the broader network of pathways modulated by SGLT2i therapy.

High-throughput methods such as bulk and scRNA-seq now allow unbiased profiling of SGLT2-dependent transcriptional programs across nephron segments and disease states [50,51]. Meanwhile, network-based bioinformatic analyses can infer upstream regulators and interaction partners of SGLT2 and its signaling modules [23]. In parallel, epigenomic assays, including chromatin immunoprecipitation followed by sequencing, transposase-accessible chromatin-seq assay, single-nucleus chromatin accessibility profiling, and DNA methylation analyses, provide complementary information on enhancer–promoter usage and chromatin states that underlie SGLT2 expression dynamics [16,23]. Together, these approaches move the field beyond single-gene quantification and enable systems-level dissection of how SGLT2 inhibition reshapes transcriptional and epigenetic landscapes in the kidneys.

Multi-omics studies, mainly in animal models, demonstrate that SGLT2is normalize metabolic pathways, attenuate inflammatory signaling, and modulate chromatin structure [52,53]. In diabetic mice, scRNA-seq revealed that PT cells exhibit enhanced oxidative metabolism, inflammatory activation, and fibrotic signatures. Treatment with SGLT2i reversed these abnormalities, restoring fatty acid oxidation and mitochondrial gene expression while reducing macrophage-mediated inflammation and epithelial–mesenchymal transition markers [51].

Epigenetic mechanisms further contribute to these renoprotective effects. In models of metabolic stress, SGLT2 inhibition increased renal levels of S-adenosylmethionine, promoting histone H3 lysine 27 trimethylation in NF-κB-related genes and repressing inflammatory transcription [54]. Inhibition of the S-adenosylmethionine synthetase MAT2A abrogated these protective effects, linking SGLT2 loss to methyl-donor-dependent epigenetic remodeling [54]. These findings provide mechanistic evidence that SGLT2 inhibition stabilizes the tubular epigenome and restrains maladaptive gene programs.

Beyond diabetes, SGLT2is also ameliorate transcriptomic signatures of fibrosis in hyperuricemic and hypertensive models. Activation of the estrogen-related receptor-α/organic anion transporter-1 axis enhances uric acid excretion and attenuates tubulointerstitial fibrosis [55]. Meanwhile, in hypertensive rats, broad RNA-seq profiling has identified renal, adipose, and pulmonary tissues as major sites of transcriptional response [50]. scRNA-seq of kidney tissues from diabetic mice revealed that SGLT2i directly attenuates PT fibrosis by downregulating 3-hydroxy-3-methylglutaryl-CoA synthase 2 in PT cells and reducing a fibrosis-associated tubular subpopulation marked by high B-cell translocation gene 2 expression. Moreover, SGLT2is indirectly modulate macrophage inflammation through altered epithelial–macrophage communication, particularly via diminished App–CD74 signaling [56]. Dapagliflozin also modulates PT metabolism in diabetic kidneys by reducing excessive oxidative phosphorylation and improving tissue oxygenation. In PT cells from diabetic mice, RNA sequencing revealed significant downregulation of oxidative phosphorylation pathways after 8 weeks of dapagliflozin treatment. Despite this transcriptional reduction, dapagliflozin ameliorated the energetic depletion and hypoxia in renal tissue observed in untreated diabetic mice [57], suggesting that hypoxic stress relief represents another key mechanism contributing to the renoprotective effects of SGLT2 inhibition in diabetic kidney disease.

**Figure 2:**
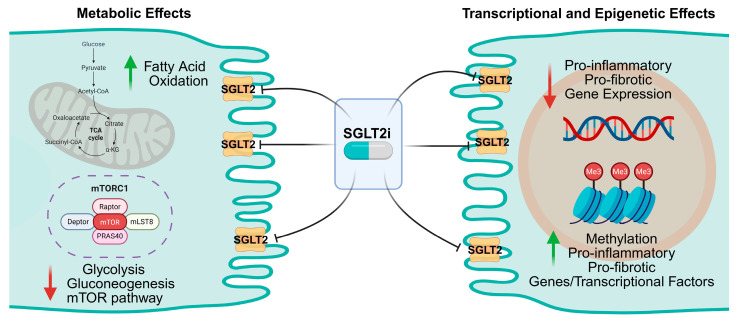
**Metabolic, transcriptional, and epigenetic effects of sodium–glucose cotransporter 2 inhibitors in the proximal tubule.** The figure summarizes the key molecular adaptations induced by sodium–glucose cotransporter 2 (SGLT2) inhibition, including transcriptional reprogramming, metabolic pathway modulation, such as reduced glycolysis and tricarboxylic acid cycle (TCA) activity, and suppression of mechanistic target of rapamycin complex 1 (mTORC1) signaling. Epigenetic remodeling further supports enhanced energy efficiency and reduced cellular stress. SGLT2i: sodium–glucose cotransporter 2 inhibitors.

These molecular adaptations involve pathways regulating mitochondrial function, adenosine monophosphate-activated protein kinase signaling, and inflammation, suggesting that the genomic footprint of SGLT2 inhibition extends across metabolic and vascular systems [50,55]. Collectively, animal and in vitro studies demonstrate that SGLT2is modulate both gene transcription and chromatin state, promoting energy-efficient metabolism and reducing cellular stress, as summarized in Figure 2. However, until recently, these mechanisms had not been directly examined in human kidney tissue.

### Human Data: The Only scRNA-seq Study to Date

The most comprehensive human evidence to date comes from a landmark study that combined scRNA-seq and morphometric analyses of kidney biopsies from young individuals with T2DM and healthy controls [42]. To our knowledge, this investigation represents the only currently available human kidney-biopsy scRNA-seq dataset assessing the molecular effects of SGLT2 inhibition in vivo. Participants with diabetes were obese and exhibited glomerular hyperfiltration, with increased mesangial and glomerular volumes relative to controls. Among those with diabetes, 10 individuals were treated with SGLT2i, and 6 were untreated [42].

At the single-cell level, SLC5A2 transcripts localized exclusively to PT cells, with the highest expression observed in untreated kidneys. However, transcriptional alterations associated with SGLT2is were observed across multiple nephron segments, most prominently in the distal nephron. In the PT, SGLT2is suppressed glycolysis, gluconeogenesis, and tricarboxylic acid cycle pathways, while the thick ascending limb exhibited compensatory upregulation of these metabolic routes, reflecting coordinated, segment-specific energy reprogramming in response to therapy [42].

Crucially, pathway analysis revealed normalization of mechanistic target of rapamycin complex 1 signaling in the kidneys of SGLT2i-treated patients, consistent with the metabolic restoration observed in experimental models. Immunohistochemical validation demonstrated decreased phosphorylation of S6 protein in both proximal and distal tubules of treated patients, confirming suppression of mechanistic target of rapamycin complex 1 activity [42]. These findings establish a direct molecular link between SGLT2 inhibition and improved tubular energy homeostasis in human diabetes, providing translational confirmation of the mechanistic hypotheses derived from preclinical research.

The molecular signals underlying the transcriptional and epigenetic effects of SGLT2 inhibition in the kidneys are listed in Table 1.

## 4. Knowledge Gaps and Future Directions

Despite major advances, several aspects of SGLT2 biology remain unresolved. First, integrated multi-omics profiling of human kidneys exposed to SGLT2i remains limited. Epigenomic mapping through assay for transposase-accessible chromatin sequencing or single-nucleus chromatin accessibility assays remains rare, with most evidence derived from histone-mark profiling rather than open-chromatin analysis. Whole-genome methylome studies in the context of SGLT2 inhibition are also lacking, leaving uncertainty about direct DNA methylation effects. Spatial transcriptomic approaches could further clarify how SGLT2is reshape local metabolic gradients and intercellular signaling within the nephron.

Second, the temporal sequence of transcriptional and metabolic adaptations following SGLT2i initiation is unknown. Longitudinal human studies combining functional imaging with molecular sampling could determine whether suppression of mechanistic target of rapamycin complex 1 activity precedes clinical improvements in kidney function. Third, the interplay between SGLT2 inhibition and other glucose transporters, such as sodium–glucose cotransporter 1 and glucose transporter 2, warrants deeper investigation, particularly concerning compensatory mechanisms and cross-regulation.

Finally, the impact of patient heterogeneity—including age, sex, obesity, and genetic background—on SGLT2 expression and therapeutic responsiveness has not been systematically examined but is likely to be critical to the development of precision medicine approaches. Addressing these gaps through integrative, human-focused research will be essential to fully elucidate the renoprotective mechanisms of SGLT2is and refine precision therapeutic strategies.

## 5. Conclusions

Renal SGLT2 is a finely regulated glucose transporter whose expression reflects both systemic metabolic status and local tubular integrity. In health, it maintains glucose and sodium balance through PT reabsorption, governed by hormonal, neural, and epigenetic signals. In disease, SGLT2 expression fluctuates dynamically, rising in early diabetes and obesity but declining with progressive fibrosis. Experimental and clinical transcriptomic data demonstrate that pharmacological inhibition of SGLT2 extends beyond blockade of glucose transport, encompassing metabolic reprogramming and suppression of mechanistic target of rapamycin complex 1 signaling. The only human scRNA-seq study to date provides compelling evidence that SGLT2i therapy restores tubular energy balance and mitigates diabetes-induced metabolic stress. Future studies employing integrated multi-omics and spatial analyses will be crucial to confirm these mechanisms, define causal pathways, and harness the full therapeutic potential of SGLT2 inhibition in kidney disease.

## Figures and Tables

**Table 1:** Molecular signals underlying transcriptional and epigenetic effects of sodium–glucose cotransporter 2 inhibition in the kidneys.

Model	Omics	Exposure	Key Molecular Signals	Post-Intervention Findings
Human kidneys (adolescents with T2DM ^17^)	scRNA-seq ^14^	On SGLT2i ^16^ vs. no SGLT2i ^16^ (in vivo)	Tubular metabolic rewiring; ↓mTORC1 ^10^ signatures; shifts in stress response pathways [42]	SGLT2is ^16^ attenuate transcriptional signs of altered renal metabolism
Mouse DKD ^5^	scRNA-seq ^14^	SGLT2i ^16^ vs. ARB ^1^	Restoration of mitochondrial FAO ^6^ programs [51]	SGLT2is ^16^ mainly affect mitochondrial function in PT ^11^ cells; ARB ^1^ mostly has anti-inflammatory and anti-fibrotic effects [51]
Mouse kidneys ± human PT ^11^	RNA-seq ^12^ CUT&RUN	Genetic/pharmacologic SGLT2 ^15^ loss/inhibition	↑SAM ^13^ and ↑H3K27me3 ^7^ at the inflammatory loci; repression of inflammatory gene programs [54]	Absence of SGLT2 ^15^ induces protective methylation in the kidneys; SGLT2i ^16^ mimics anti-inflammatory effects of genetic SGLT2 ^15^ loss [54]
Mouse multi-tissue (including the kidneys)	Bulk RNA-seq ^12^	Empagliflozin	↓MAPK10 ^9^ as a key DEG ^4^; the kidney is the tissue most affected by the transcriptional changes [50]	Empagliflozin influences gene and protein expression across different tissues [50]
Mouse DKD ^5^	scRNA-seq ^14^	SGLT2i ^16^	Anti-fibrotic shifts; tubular cell-state remodeling [56]	SGLT2is ^16^ exert anti-inflammatory effects by modulating cell–cell communication; Hmgcs2 ^8^ and Btg2 ^3^ are SGLT2i ^16^ targets to ameliorate fibrosis in DKD ^5^ [56]
Mouse DKD ^5^	Bulk RNA-seq ^12^	Dapagliflozin	DEG ^4^ in metabolism, energy production, and hypoxia-related stress in PT ^11^ cells [57]	Dapagliflozin decreases oxygen and ATP ^2^ consumption in diabetic kidneys [57]

^1^ ARB: angiotensin receptor blocker; ^2^ ATP: adenosine triphosphate; ^3^ Btg2: B-cell translocation gene 2; ^4^ DEG: differentially expressed gene; DKD: ^5^ diabetic kidney disease; ^6^ FAO: fatty acid oxidation; ^7^ H3K27me3: trimethylated histone H3 lysine 27; ^8^ Hmgcs2: 3-hydroxy-3-methylglutaryl-CoA synthase 2; ^9^ MAPK10: mitogen-activated protein kinase 10; ^10^ mTORC1: mechanistic target of rapamycin complex 1; ^11^ PT: proximal tubule; ^12^ RNA-seq: RNA sequencing; ^13^ SAM: S-adenosylmethionine; ^14^ scRNA-seq: single-cell RNA sequencing; ^15^ SGLT2: sodium–glucose cotransporter 2; ^16^ SGLT2i: sodium–glucose cotransporter 2 inhibitor; ^17^ T2DM: type 2 diabetes mellitus. ↑, increase, ↓, decrease.

## Data Availability

No new data were created or analyzed in this study. Data sharing does not apply to this article.

## Abbreviations

The following abbreviations were used in this manuscript:

Ang II	Angiotensin II
ARB	Angiotensin receptor blocker
ATP	Adenosine triphosphate
BP	Blood pressure
Btg2	B-cell translocation gene 2
cAMP	3′,5′-cyclic adenosine monophosphate
CCKBR	Cholecystokinin B receptor
CKD	Chronic kidney disease
DEG	Differentially expressed gene
DKD	Diabetic kidney disease
FAO	Fatty acid oxidation
H3K27me3	Trimethylated histone H3 lysine 27
Hmgcs2	3-hydroxy-3-methylglutaryl-CoA synthase 2
IL-6	Interleukin-6
MAPK10	Mitogen-activated protein kinase 10
mTORC1	Mechanistic target of rapamycin complex 1
NF-κB	Nuclear factor-kappa B
PCR	Polymerase chain reaction
PPARδ	Peroxisome proliferator-activated receptor-δ
PT	Proximal convoluted tubule
RAAS:	Renin–angiotensin–aldosterone system
RNA-seq	RNA sequencing
RT-qPCR	Real-time quantitative polymerase chain reaction
SAM	S-adenosylmethionine
scRNA-seq	Single-cell RNA sequencing
SGLT2	Sodium–glucose cotransporter 2
SGLT2i	Sodium–glucose cotransporter 2 inhibitors
SNS	Sympathetic nervous system
T2DM	Type 2 diabetes mellitus
TCA	Tricarboxylic acid cycle
TGF-β	Transforming growth factor-β
TNF-α	Tumor necrosis factor-α
YB-1	Y-box binding protein-1

## References

[1] Giugliano D., Longo M., Scappaticcio L., Bellastella G., Maiorino M.I., Esposito K. (2021). SGLT-2 Inhibitors and Cardiorenal Outcomes in Patients with or without Type 2 Diabetes: A Meta-Analysis of 11 CVOTs. Cardiovasc. Diabetol..

[2] Armillotta M., Angeli F., Paolisso P., Belmonte M., Raschi E., Di Dalmazi G., Amicone S., Canton L., Fedele D., Suma N. (2025). Cardiovascular Therapeutic Targets of Sodium-Glucose Co-Transporter 2 (SGLT2) Inhibitors beyond Heart Failure. Pharmacol. Ther..

[3] Perkovic V., Jardine M.J., Neal B., Bompoint S., Heerspink H.J.L., Charytan D.M., Edwards R., Agarwal R., Bakris G., Bull S. (2019). Canagliflozin and Renal Outcomes in Type 2 Diabetes and Nephropathy. N. Engl. J. Med..

[4] The EMPA-KIDNEY Collaborative Group (2023). Empagliflozin in Patients with Chronic Kidney Disease. N. Engl. J. Med..

[5] Heerspink H.J.L., Stefánsson B.V., Correa-Rotter R., Chertow G.M., Greene T., Hou F.-F., Mann J.F.E., McMurray J.J.V., Lindberg M., Rossing P. (2020). Dapagliflozin in Patients with Chronic Kidney Disease. N. Engl. J. Med..

[6] Baigent C., Emberson J., Haynes R., Herrington W.G., Judge P., Landray M.J., Mayne K.J., Ng S.Y.A., Preiss D., Roddick A.J. (2022). Impact of Diabetes on the Effects of Sodium Glucose Co-Transporter-2 Inhibitors on Kidney Outcomes: Collaborative Meta-Analysis of Large Placebo-Controlled Trials. Lancet.

[7] Neuen B.L., Fletcher R.A., Anker S.D., Bhatt D.L., Butler J., Cherney D.Z.I., Docherty K.F., Inzucchi S.E., Jardine M.J., Mahaffey K.W. (2025). SGLT2 Inhibitors and Kidney Outcomes by Glomerular Filtration Rate and Albuminuria. JAMA.

[8] Staplin N., Roddick A.J., Neuen B.L., Anker S.D., Bhatt D.L., Butler J., Cherney D.Z., Docherty K.F., Fletcher R.A., Inzucchi S.E. (2025). Effects of Sodium Glucose Cotransporter 2 Inhibitors by Diabetes Status and Level of Albuminuria. JAMA.

[9] Ferreira J.P., Marques P., Anker S.D., Butler J., Filippatos G., Sharma A., Vasques-Nóvoa F., Mendonça L., Neves J.S., Packer M. (2025). Effects of SGLT2 Inhibitors across the Spectrum of Albuminuria in Cardiovascular–Kidney–Metabolic Conditions: A Pooled Analysis of Randomised Trials. Diabetes Obes. Metab..

[10] Pucci C., Marazza D.S., Preka E., Mastrangelo A., Montini G., Boyer O. (2025). SGLT2 Inhibitors for Kidney Protection in Children: Expanding Horizons beyond Endocrinology. Pediatr. Nephrol..

[11] Sarzani R., Giulietti F., Di Pentima C., Spannella F. (2020). Sodium-Glucose Co-Transporter-2 Inhibitors: Peculiar “Hybrid” Diuretics That Protect from Target Organ Damage and Cardiovascular Events. Nutr. Metab. Cardiovasc. Dis..

[12] Radlinger B., Ress C., Folie S., Salzmann K., Lechuga A., Weiss B., Salvenmoser W., Graber M., Hirsch J., Holfeld J. (2023). Empagliflozin Protects Mice against Diet-Induced Obesity, Insulin Resistance and Hepatic Steatosis. Diabetologia.

[13] Lee Y.-H., Lim S., Davies M.J. (2025). Cardiometabolic and Renal Benefits of Sodium–Glucose Cotransporter 2 Inhibitors. Nat. Rev. Endocrinol..

[14] Vallon V. (2024). How Can Inhibition of Glucose and Sodium Transport in the Early Proximal Tubule Protect the Cardiorenal System?. Nephrol. Dial. Transplant..

[15] Wu Q., Zhang J., Zhang F., Li D. (2025). SGLT2 Inhibitors as Metabolic Modulators: Beyond Glycemic Control in Type 2 Diabetes. Front. Endocrinol..

[16] Mao Z.-H., Gao Z.-X., Liu Y., Liu D.-W., Liu Z.-S., Wu P. (2023). Single-Cell Transcriptomics: A New Tool for Studying Diabetic Kidney Disease. Front. Physiol..

[17] Wright E.M., Loo D.D.F., Hirayama B.A. (2011). Biology of Human Sodium Glucose Transporters. Physiol. Rev..

[18] Vallon V., Platt K.A., Cunard R., Schroth J., Whaley J., Thomson S.C., Koepsell H., Rieg T. (2011). SGLT2 Mediates Glucose Reabsorption in the Early Proximal Tubule. J. Am. Soc. Nephrol..

[19] Xu L., Zhao R., Zhao Y., Tang X., Si N., Guo X., Yue C., Nie M., Chen L. (2024). Genetic and Clinical Characterization of Familial Renal Glucosuria. Clin. Kidney J..

[20] Muto Y., Wilson P.C., Ledru N., Wu H., Dimke H., Waikar S.S., Humphreys B.D. (2021). Single Cell Transcriptional and Chromatin Accessibility Profiling Redefine Cellular Heterogeneity in the Adult Human Kidney. Nat. Commun..

[21] Ghezzi C., Loo D.D.F., Wright E.M. (2018). Physiology of Renal Glucose Handling via SGLT1, SGLT2 and GLUT2. Diabetologia.

[22] Vallon V. (2020). Glucose Transporters in the Kidney in Health and Disease. Pflug. Arch..

[23] Wicik Z., Nowak A., Jarosz-Popek J., Wolska M., Eyileten C., Siller-Matula J.M., von Lewinski D., Sourij H., Filipiak K.J., Postuła M. (2022). Characterization of the SGLT2 Interaction Network and Its Regulation by SGLT2 Inhibitors: A Bioinformatic Analysis. Front. Pharmacol..

[24] Jarosz-Popek J., Eyileten C., Gager G.M., Nowak A., Szwed P., Wicik Z., Palatini J., von Lewinski D., Sourij H., Siller-Matula J.M. (2024). The Interaction between Non-Coding RNAs and SGLT2: A Review. Int. J. Cardiol..

[25] Takesue H., Hirota T., Tachimura M., Tokashiki A., Ieiri I. (2018). Nucleosome Positioning and Gene Regulation of the SGLT2 Gene in the Renal Proximal Tubular Epithelial Cells. Mol. Pharmacol..

[26] Bansal A., Chonchol M. (2025). Metabolic Dysfunction–Associated Kidney Disease: Pathogenesis and Clinical Manifestations. Kidney Int..

[27] Kanbay M., Copur S., Guldan M., Ozbek L., Hatipoglu A., Covic A., Mallamaci F., Zoccali C. (2024). Proximal Tubule Hypertrophy and Hyperfunction: A Novel Pathophysiological Feature in Disease States. Clin. Kidney J..

[28] Lee-Boey J.-W.S., Tan J.-K., Lim Z.-F., Zaccardi F., Khunti K., Ezzati M., Gregg E.W., Lim L.-L. (2025). Obesity-related Glomerulopathy: How It Happens and Future Perspectives. Diabet. Med..

[29] Nakamura N., Matsui T., Ishibashi Y., Yamagishi S.-I. (2015). Insulin Stimulates SGLT2-Mediated Tubular Glucose Absorption via Oxidative Stress Generation. Diabetol. Metab. Syndr..

[30] Pochynyuk O., Pyrshev K., Cheng X. (2025). Multifaceted Roles of Epac Signaling in Renal Functions. Biochem. J..

[31] Beloto-Silva O., Machado U.F., Oliveira-Souza M. (2011). Glucose-Induced Regulation of NHEs Activity and SGLTs Expression Involves the PKA Signaling Pathway. J. Membr. Biol..

[32] Zhang X., Zhang Y., Shi Y., Shi D., Niu M., Liu X., Liu X., Yang Z., Wu X. (2025). Kidney Gastrin/CCKBR Attenuates Type 2 Diabetes Mellitus by Inhibiting SGLT2-Mediated Glucose Reabsorption through Erk/NF-ΚB Signaling Pathway. Diabetes Metab. J..

[33] Maldonado-Cervantes M.I., Galicia O.G., Moreno-Jaime B., Zapata-Morales J.R., Montoya-Contreras A., Bautista-Perez R., Martinez-Morales F. (2012). Autocrine Modulation of Glucose Transporter SGLT2 by IL-6 and TNF-α in LLC-PK1 Cells. J. Physiol. Biochem..

[34] Umino H., Hasegawa K., Minakuchi H., Muraoka H., Kawaguchi T., Kanda T., Tokuyama H., Wakino S., Itoh H. (2018). High Basolateral Glucose Increases Sodium-Glucose Cotransporter 2 and Reduces Sirtuin-1 in Renal Tubules through Glucose Transporter-2 Detection. Sci. Rep..

[35] Miyata K.N., Lo C.-S., Zhao S., Liao M.-C., Pang Y., Chang S.-Y., Peng J., Kretzler M., Filep J.G., Ingelfinger J.R. (2021). Angiotensin II Up-Regulates Sodium-Glucose Co-Transporter 2 Expression and SGLT2 Inhibitor Attenuates Ang II-Induced Hypertensive Renal Injury in Mice. Clin. Sci..

[36] Matthews V.B., Elliot R.H., Rudnicka C., Hricova J., Herat L., Schlaich M.P. (2017). Role of the Sympathetic Nervous System in Regulation of the Sodium Glucose Cotransporter 2. J. Hypertens..

[37] Herat L.Y., Magno A.L., Rudnicka C., Hricova J., Carnagarin R., Ward N.C., Arcambal A., Kiuchi M.G., Head G.A., Schlaich M.P. (2020). SGLT2 Inhibitor–Induced Sympathoinhibition. JACC Basic Transl. Sci..

[38] Chen J., Li T., Vladmir C., Yuan Y., Sun Z. (2021). Renal Lipid Accumulation Induced by High-Fat Diet Regulates Glucose Homeostasis via Sodium-Glucose Cotransporter 2. Diabetes Res. Clin. Pract..

[39] Zhao Y., Gao P., Sun F., Li Q., Chen J., Yu H., Li L., Wei X., He H., Lu Z. (2016). Sodium Intake Regulates Glucose Homeostasis through the PPARδ/Adiponectin-Mediated SGLT2 Pathway. Cell Metab..

[40] Bernhardt A., Häberer S., Xu J., Damerau H., Steffen J., Reichardt C., Wolters K., Steffen H., Isermann B., Borucki K. (2021). High Salt Diet-induced Proximal Tubular Phenotypic Changes and Sodium-glucose Cotransporter-2 Expression Are Coordinated by Cold Shock Y-box Binding Protein-1. FASEB J..

[41] Jia H., Bao P., Yao S., Zhang X., Mu J.-J., Hu G.-L., Du M.-F., Chu C., Zhang X.-Y., Wang L. (2023). Associations of SGLT2 Genetic Polymorphisms with Salt Sensitivity, Blood Pressure Changes and Hypertension Incidence in Chinese Adults. Hypertens. Res..

[42] Schaub J.A., AlAkwaa F.M., McCown P.J., Naik A.S., Nair V., Eddy S., Menon R., Otto E.A., Demeke D., Hartman J. (2023). SGLT2 Inhibitors Mitigate Kidney Tubular Metabolic and MTORC1 Perturbations in Youth-Onset Type 2 Diabetes. J. Clin. Investig..

[43] Srinivasan Sridhar V., Ambinathan J.P.N., Kretzler M., Pyle L.L., Bjornstad P., Eddy S., Cherney D.Z., Reich H.N., European Renal cDNA Bank (ERCB) (2019). Nephrotic Syndrome Study Network (NEPTUNE) Renal SGLT MRNA Expression in Human Health and Disease: A Study in Two Cohorts. Am. J. Physiol. Renal Physiol..

[44] Youm E.B., Shipman K.E., Albalawy W.N., Vandevender A.M., Sipula I.J., Rbaibi Y., Marciszyn A.E., Lashway J.A., Brown E.E., Bondi C.B. (2024). Megalin Knockout Reduces SGLT2 Expression and Sensitizes to Western Diet-Induced Kidney Injury. Function.

[45] Korsten P., Tampe B. (2023). A Transcriptome Array-Based Approach to Link SGLT-2 and Intrarenal Complement C5 Synthesis in Diabetic Nephropathy. Int. J. Mol. Sci..

[46] Allahwala M.A., Marathe C.S., Nelson A.J., Psaltis P.J., Marathe J.A. (2025). Established and Emerging Therapies for Cardiovascular-Kidney-Metabolic Syndrome: Harnessing the Benefits of SGLT-2 Inhibitors, GLP-1 Receptor Agonists, and Beyond. Heart Lung Circ..

[47] Mohamed H.E., Asker M.E., Keshawy M.M., Hasan R.A., Mahmoud Y.K. (2020). Inhibition of Tumor Necrosis Factor-α Enhanced the Antifibrotic Effect of Empagliflozin in an Animal Model with Renal Insulin Resistance. Mol. Cell. Biochem..

[48] Cortés-Camacho F., Zambrano-Vásquez O.R., Aréchaga-Ocampo E., Castañeda-Sánchez J.I., Gonzaga-Sánchez J.G., Sánchez-Gloria J.L., Sánchez-Lozada L.G., Osorio-Alonso H. (2024). Sodium–Glucose Cotransporter Inhibitors: Cellular Mechanisms Involved in the Lipid Metabolism and the Treatment of Chronic Kidney Disease Associated with Metabolic Syndrome. Antioxidants.

[49] Koch B., Fuhrmann D.C., Schubert R., Geiger H., Speer T., Baer P.C. (2023). Gliflozins Have an Anti-Inflammatory Effect on Renal Proximal Tubular Epithelial Cells in a Diabetic and Inflammatory Microenvironment In Vitro. Int. J. Mol. Sci..

[50] Tan F., Long X., Du J., Yuan X. (2023). RNA-Seq Transcriptomic Landscape Profiling of Spontaneously Hypertensive Rats Treated with a Sodium-Glucose Cotransporter 2 (SGLT2) Inhibitor. Biomed. Pharmacother..

[51] Wu J., Sun Z., Yang S., Fu J., Fan Y., Wang N., Hu J., Ma L., Peng C., Wang Z. (2022). Kidney Single-Cell Transcriptome Profile Reveals Distinct Response of Proximal Tubule Cells to SGLT2i and ARB Treatment in Diabetic Mice. Mol. Ther..

[52] Wu H., Gonzalez Villalobos R., Yao X., Reilly D., Chen T., Rankin M., Myshkin E., Breyer M.D., Humphreys B.D. (2022). Mapping the Single-Cell Transcriptomic Response of Murine Diabetic Kidney Disease to Therapies. Cell Metab..

[53] Wolf L., Föller M., Feger M. (2023). The Impact of SGLT2 Inhibitors on AKlotho in Renal MDCK and HK-2 Cells. Front. Endocrinol..

[54] Maekawa H., Zhou Y., Aoi Y., Fain M.E., Kaminski D.S., Kong H., Sebo Z.L., Chakrabarty R.P., Howard B.C., Andersen G. (2025). SGLT2 Inhibition Protects Kidney Function by SAM-Dependent Epigenetic Repression of Inflammatory Genes under Metabolic Stress. J. Clin. Investig..

[55] Hu H., Li W., Hao Y., Peng Z., Zou Z., Wei J., Zhou Y., Liang W., Cao Y. (2024). The SGLT2 Inhibitor Dapagliflozin Ameliorates Renal Fibrosis in Hyperuricemic Nephropathy. Cell Rep. Med..

[56] Yan S., Luo M., Zhou R., Peng F., Zhang M., Feng Y., Zhao L., Yang L., Cheng Y. (2025). Sodium-Glucose Cotransporter 2 Inhibitors Alleviate Renal Fibrosis in Diabetic Kidney Disease by Inhibiting Hmgcs2 and Btg2 in Proximal Tubular Cells. J. Transl. Med..

[57] Uehara-Watanabe N., Okuno-Ozeki N., Nakamura I., Nakata T., Nakai K., Yagi-Tomita A., Ida T., Yamashita N., Kamezaki M., Kirita Y. (2022). Proximal Tubular Epithelia-Specific Transcriptomics of Diabetic Mice Treated with Dapagliflozin. Heliyon.

